# Becoming eligible for long-term care insurance in China brought more ageing at home: evidence from a pilot city

**DOI:** 10.1093/heapol/czae109

**Published:** 2024-11-09

**Authors:** Zeyuan Chen, Hui Zhou, Xiang Ma

**Affiliations:** School of Public Administration, Southwestern University of Finance and Economics, Liutai Avenue 555, Chengdu 611130, China; Cao County Tax Service, State Taxation Administration, Gongye Road 230, Cao County, Heze 274000, China; School of Economics, Southwestern University of Finance and Economics, Liutai Avenue 555, Chengdu 611130, China

**Keywords:** long-term care insurance, event study, aging in place, opportunity cost

## Abstract

Person-centered long-term care systems, integral to healthy ageing, should empower older people to achieve ageing in place. Yet evidence on the impact of the design of long-term care systems on older people’s choice of place of ageing, especially that from developing countries, is limited. Taking the introduction of Long-Term Care Insurance (LTCI) in City X of China as a policy shock, we examined the impact of becoming eligible for LTCI on program beneficiaries’ choice of place of ageing—institution or home—before they started to receive any actual benefit. Based on our analysis of the administrative data of all LTCI applicants between July 2017 and September 2020 from City X, we found that becoming eligible for LTCI increased an older-person’s probability of choosing home as her place of ageing even before she received any benefit by ∼16%, and this positive impact was larger for those insured, of higher education level, or of higher disability grade. By bringing more ageing in place, LTCI in City X promoted healthy ageing. Our study suggests that the specifics of the LTCI program, such as who could receive subsidies, family values, and family members’ engagement in the labor market, could all work together to shape the substitution pattern between home and institutional care.

**Key messages**

- Ageing in place, aligned well with older people’s preference, improves their subjective well-being and contributes to healthy ageing.
- More people moved to home care once becoming eligible for LTCI in a pilot city of China, even before they started to receive any actual benefit.
- Covering informal home care alongside formal care by LTCI could moderate the conflict between traditional filial piety and the modern market-based economy, and encourage ageing in place.

## Introduction

The rapidly ageing population poses a great challenge to the world. Older people, often with physical or mental disabilities, traditionally rely on informal home care. However, this tradition becomes unsustainable as family size decreases, female labor-force participation increases, and migration across regions grows (Campbell and Ikegami 2000). In response to this challenge, a number of countries have introduced long-term care (LTC) services to compensate for insufficient informal family care (Campbell and Ikegami 2000, Chen et al. 2020). Depending on the structure of their social security systems, different countries arrange their LTC in different ways. In the USA, while two main public programs, Medicare and Medicaid, pay for ∼60% of formal LTC costs, most home care is still provided informally by relatives (Gruber and McGarry 2023). In welfare states such as Sweden, the LTC system is decentralized, and municipalities are responsible for LTC services. They mainly cover institutional care and formal home care, while cash benefits for family caregivers play a marginal role (Schön and Josephine 2018). In Germany, LTC insurance (LTCI) covers institutional care, formal home care, and cash allowances for informal care (Geyer and Korfhage 2015). In Japan and Korea, LTCI covers institutional care and formal home care (Campbell and Ikegami 2000, Kim and Lim 2015). While Germany established its LTCI in 1995 (Geyer and Korfhage 2015), Asian countries like Japan and Korea introduced their LTCI in 2000 and 2008, respectively (Campbell and Ikegami 2000, Kim and Lim 2015).

Though originally designed to encourage formal institutional care, LTC services in practice and policy emphasis have increasingly shifted towards home and community-based care (Colombo et al. 2011, Feng et al. 2020b). This shift partly reveals older people’ preference to age in their homes or communities (Binette and Farago 2021, Feng et al. 2020b), driven by their demand for a sense of connection, security, familiarity, and identity (Wiles et al. 2012). This calls for building person-centered LTC systems aligned with older people’s values and preferences to promote healthy ageing in place (World Health Organization 2015, 2021a, 2021b).

In 2021, China had 267.4 million people aged ≥60 years, accounting for 18.9% of its total population (Department of Older Adults’ Health 2022). A fair portion of them had disabilities or health conditions (Xinhua News Agency 2016). Data from the 2020 Population Census showed that of those aged ≥60 years, 12.8% rated themselves as unhealthy and 2.3% were too unhealthy to be able to take care of themselves (Office of the Leading Group of the State Council for the Seventh National Population Census 2022). Similarly, data from the 2020 special wave of China Health and Retirement Longitudinal Study (CHARLS) showed that of those aged ≥60 years, 17.8% required assistance with at least one activity of daily living or an instrumental activity of daily living (Gong et al. 2022).

In order to learn how to cope with the ageing problem, China introduced LTCI in 15 pilot cities in 2017 (supplementary Table S1, see online supplementary material). Before the pilot program of LTCI, public support for LTC was minimal and was mostly limited to welfare recipients who typically had no child or other family member to care for them (Feng et al. 2020b). For those who were classified as ‘Three No’s’ (no ability to work, no source of income, and no family support) in urban areas and ‘Five Guarantees’ (government guarantees food, clothing, housing, medical care, and burial expenses) in rural areas, the government provided living costs and LTC support. In 2014, ‘Three No’s’ and ‘Five Guarantees’ accounted for only 2.5% of adults aged >60 years (Feng et al. 2020b). Regarding private insurance, data from the 2018 wave of CHARLS showed that only 3.6% of the respondents (731 out of 19 772) had any private medical insurance, and only 0.1% (20 out of 19 772) reported any LTCI. By 2021, the pilot had expanded to 49 cities and covered 144.6 million insured people (Department of Older Adults’ Health 2022). A challenge in designing the LTCI program that China had to face was its older people’s strong preference for informal family care. Data from the Fourth National Survey on Older People’ Living Conditions in Urban/Rural China conducted in 2015 showed that of those older people who were unable to take care of themselves, 89.9% preferred to receive care in their homes, while only 4.7% preferred to receive care at older-people care institutions (Chen 2018). The strong preference for informal family care in China partly arises from its distinct Confucian culture, where filial piety (*xiao*) is a fundamental tenet. Filial piety obligates children to support and obey their parents (Ikels 2004, Chou 2011). A violation of filial piety by children would be sending their parents to older-people care institutions: these parents would feel ashamed because it signals a lack of care from their offspring (Zhan et al. 2011, Chen and Han 2016, Chen 2018).

Aligned well with older people’s strong preference for home care, Shanghai local government first proposed, and China’s central government then promoted, a ‘90-7-3’ structure for its LTC services, with 90% of older people receiving care in their homes, 7% supported by community-based services, and 3% in institutions (Chen et al. 2022, Chen and Han 2016, Feng et al. 2020b). However, there is a concern that actual policy implementation might have instead incentivized a disproportionate growth of institutional care relative to home or community-based care, contrary to older people’s strong preference for family care and the original governmental plan (Feng et al. 2020b). Few studies to date have examined the impact of China’s pilot LTCI on older people’ choice of place of ageing. Most studies of the pilot LTCI in China examined its impact on care recipients’ hospitalization and medical expenditure (Feng et al. 2020a, Lu et al. 2020, Chen and Ning 2022, Liu et al. 2023b), health status (Wang et al. 2023, Zhou et al. 2023), consumption (Liu et al. 2023a), wellbeing (Lei et al. 2022), or caregivers’ health and well-being (Chen et al. 2019, Ai et al. 2022).

In this study we examined the impact of the eligibility for LTCI on program beneficiaries’ choice of place of ageing—institution or home—even before they started to receive any actual benefit, using the administrative data of all LTCI applicants between July 2017 and September 2020 from one of the pilot cities, City X in China. In practice, each pilot city designed its own LTCI program (supplementary Table S1). The LTCI in City X targeted older people with disabilities. Differing from most other pilot cities, City X provided benefits not only to institutions, but also directly to family caregivers. We employed the introduction of LTCI as an external shock and adopted both an event study approach and a difference-in-differences (DID) method to identify the impact.

### Policy background

City X possesses a population of ∼15 million. In 2017, the average disposable income for its urban residents was close to the country’s average, 36 396 CNY (about 5721 USD) (National Bureau of Statistics of China 2018). In this sense, City X could be treated as a representative middle-income city in China. City X launched its pilot on 1 July 2017. In the first phase, the LTCI covered those who were beneficiaries of Urban Employee Basic Medical Insurance (UEBMI). Among these beneficiaries, those who had been diagnosed with disabilities for at least 6 months were entitled to apply for the benefit (see supplementary Fig. S1 in the online supplementary material for the application process). After application, the government entrusted professionals to assess each applicant’s severity of disability. Then according to the assessment scores, they classified all applicants into three groups—people with mild (65 ≤ score < 100), moderate (45 ≤ score ≤ 60), and heavy (score ≤ 40) disabilities. According to the assessment rule, every score could only be a multiple of five. Only those with heavy disabilities were eligible for the benefit and they were further categorized into three grades—people with grade-one, grade-two, and grade-three heavy disabilities—and grade-three was the most severe group. Details of the assessment procedure and the criteria are provided in supplementary Note 1 (see online supplementary material). Given the objective and professional assessment of severity of disability, those being assessed were unlikely to be able to manipulate their health conditions in the on-site evaluation.

The local government officers believed that the LTCI should compensate caregivers in general, whether they were family members or institutional care providers. This rationale led the LTCI in City X to cover informal home care alongside formal institutional care in the first phase, and further extend the coverage to professional home care services in the second phase. The local government of City X did not decide who was qualified for home or institutional care. Instead, the insured themselves or their family members made the choice. The levels of monthly benefit, which differed by place of ageing (institution or home) and grade of disability, were settled at the beginning by the LTCI fund. In order to encourage ageing in place, the benefit for home care (1077, 1437, and 1796 CNY, equivalent to 169, 226, and 282 USD per month, for those with grade-one, grade-two, and grade-three heavy disabilities, respectively) was slightly higher than that of institutional care (1006, 1341, and 1676 CNY, equivalent to 158, 211, and 263 USD per month, for those with grade-one, grade-two, and grade-three heavy disabilities, respectively) in the first phase.

The second phase started in July 2020. The policy ceased to prioritize home care and raised the benefit of institutional care to be the same as that of home care (1377, 1837, and 2296 CNY, equivalent to 200, 266, and 333 USD per month for those with grade-one, grade-two, and grade-three heavy disabilities, respectively). Furthermore, the LTCI started to cover professional home-care services. Every month professionals provided formal home-care services several times for those who had chosen home as their place of ageing. People could choose whether to employ formal home-care or not. Family caregivers—in most cases some family members—got ∼80% of the whole benefit, with the remaining 20% saved for potential formal home care. Supplementary Table S2 (see online supplementary material) details the exact levels of benefit for the insured of each type in the second phase. Though informal family caregivers received slightly less than institutional care workers then, the local government provided professional training for them in order to improve care quality. In a follow-up survey conducted between July 2021 and January 2022 that covered all LTCI applicants in City X, 36.2% of respondents with heavy disabilities who were still alive then (6883 out of 19 015) reported that prior to applying for the LTCI, they received some allowances, mostly from local Bureau of Civil Affairs or Association for People with Disabilities. However, these allowances were minimal: the average being only 215 CNY (∼30 USD). Moreover, these allowances subsidized living costs and did not target caregivers. Those who participated in the professional training could get the full benefit for family caregivers; otherwise, they could only get 80% of the full benefit. City X provided a context for us to study older people’s choice of place of ageing when its LTCI covered both institutional and home care.

In theory, a similar level of benefit for home and institutional care caused an income effect for the insured. There are multiple reasons to suspect that the income effect was pro-home care. First, home care by family members is less prone to moral hazard. [In the above follow-up survey, out of 11 199 recipients who were mainly taken care of by their family members, 97.54% (*n* = 10 924) of their family caregivers reported that they spent 7 days per week in caring. Only 4.07% (*n* = 456) of them had a job simultaneously outside their family. This high intensity of caring suggested minimal moral hazard]. Perhaps this is why people prefer ageing in place in the first place. Second, as discussed above, older people in China have a strong preference for informal family care. Third and perhaps more importantly, the benefit would incentivize family caregivers more than institutional caregivers. When providing care services, family caregivers either sacrificed potential working opportunities outside or worked fewer hours per week if doing any job, and thus incurred an opportunity cost of losing income from the labor market (Giles and Mu 2007, Van Houtven et al. 2013, Giles et al. 2018). Previously without the benefit, family members were not guaranteed enough compensation for their services. On the other hand, filial piety obligates families to care for each other; but providing care to close family members or relatives in order to get monetary return violates filial piety. Facing this, family members might be reluctant to provide care services in the first place when their opportunity cost was high. Now the LTCI in City X, by directly compensating them, made family care more affordable.

Based on the above reasoning, we hypothesized that becoming eligible for the LTCI would shift older people with disabilities from institutions to homes, especially for those whose family caregivers were facing a greater opportunity cost.

## Methods

### Data and sample

We used the administrative data of all LTCI applicants between July 2017 and September 2020 from City X in China. The data contains each applicant’s information completed at application, including demographics, living conditions, application time, health conditions, months of disability by application, grade of disability, etc. Out of 36 363 applicants, 26 787 were classified as having heavy disabilities and therefore were entitled to the benefit. They then chose their places of ageing and signed an agreement with local agencies entrusted by the local government. The agreement data, also available to us, contains the time when the insured signed the agreement, place of ageing (home or institution) chosen, and if an institution was chosen, the institution’s name. The initial choice of place of ageing was inferred from the application data on living conditions and residential address. Those living in nursing hospitals or older-people care institutions were categorized as living in institutions and all the others as living in homes. The choice of place of ageing after one becoming eligible for but before starting to receive any actual benefit was inferred from the agreement data on care type (home or institutional care) chosen by care recipients. Only starting from the fifteenth of the next month after the insured signed the agreement, could they receive the benefit monthly. After excluding those without key information, 26 124 observations remained. They constituted our sample in the following event study.

### Measures

We employed the introduction of LTCI as an external shock and adopted both an event study and a DID approach to identify the impact.

Dependent variable—the dependent variable is a binary variable denoting the choice of home as the place of ageing. It took on the value zero for an individual if her living condition or residential address was any older-people care institution or nursing hospital, otherwise it took on the value one.

Independent variable—the independent variable of interest was a binary variable denoting whether an individual became eligible for the benefit. It took on the value zero before the individual signed the agreement and one after that.

Control variables—we controlled for individual fixed effects in regressions, which represented time-invariant observable and unobservable characteristics. We also controlled for year dummies, which represented time aggregate trends. For time-varying individual characteristics, we controlled for years an individual had been diagnosed with disabilities in the event study. This value of the individual’s first observation was directly recorded in her application form, and we added this value and the months between her applying for the benefit and signing the agreement to get the corresponding value of the second observation.

In the DID approach, the inclusion of more control observations (not-yet-treated or never-treated observations) enabled us to have more temporal variations. Therefore, besides years an individual had been diagnosed with disability, we also controlled for the individual’s marital status, grade of disability, dummy of mental health conditions, and dummy of any condition that demanded LTC. Marriage dummy took on the value one if the individual had a spouse, otherwise zero. The local government classified disabilities into seven grades: mild, grade-one moderate, grade-two moderate, grade-three moderate, grade-one heavy, grade-two heavy, and grade-three heavy. Both eligibility for LTCI and place of ageing were related to degree of disability. In fact, eligibility for LTCI was solely determined by degree of disability evaluated by professionals. By controlling for degree of disability, we controlled for the effect of degree of disability itself on the insured’s choice of place of ageing, and purged its effect from the DID estimates. The dummy of mental health conditions took on the value one if the individual reported any mental health conditions in the application form, otherwise zero. In their application forms, applicants needed to report whether they had been diagnosed with any of nearly 30 diseases. Among these reported diseases, Parkinson’s disease, fracture, cerebral infarction, and stroke were classified as conditions that demanded LTC. All the other aspects of the specification were the same as that of the event study.

### Statistical analysis

We first applied an event study approach to examine the impact of becoming eligible for the benefit on the insured’s choice of place of ageing before they started to receive any actual benefit. We defined the event date as the time when the insured signed the agreement with the local agencies. Consider the following econometric model,


(1) $$\begin{array}{*{20}{c}}
{Home\_car{e_{it}} = \gamma \cdot Insuranc{e_{it}} + \beta \cdot Contro{l_{it}} + {\alpha _i} + Yea{r_t} + {\varepsilon _{it}},}
\end{array}$$

where $i\,$denotes individual and $t\,$denotes calendar time. $Insuranc{e_{it}}$ denotes the eligibility status of individual $i\,$at calendar time $t.\,$Every insured $i\,$has two observations in our specification: one when she applied for LTCI ($Insuranc{e_{it}} = 0$) and the other when she signed the agreement ($Insuranc{e_{it}} = 1$).

The event study approach is appropriate here for two reasons. First, for most insured, the time interval between applying for the benefit and signing the agreement was short: the median was <1 month, and was within 2 months for 94% (supplementary Fig. S2, see online supplementary material). It is reasonable to assume that within such a short time there was no systematic time trend shifting the insured’s choice of place of ageing other than becoming eligible for the benefit. Second, note that all insured had not actually received any benefit by their second observation. In fact, what we studied was the impact of the eligibility for the benefit rather than that of the benefit itself. Therefore, what we measured could hardly arise from the possibility that the benefit affected the insured’s health status, which further shifted their choice of place of ageing.

As mentioned above, the LTCI policy was slightly adjusted in July 2020, which might contaminate our analysis. To address this issue, we adopted two strategies. First, we split the sample into two subsamples—one where individuals applied before July 2020 and the other after July 2020—and ran regressions separately for these two subsamples. Second, we constructed a binary variable denoting the above policy adjustment. It took on the value one if one applied after July 2020 and zero otherwise. We controlled for both this variable and its interaction with the independent variable of interest in regressions. For robustness, we drop those observations for time intervals between applying for the benefit and signing the agreement >100 days. To analyze heterogeneity, we ran several regressions by interacting the independent variable of interest with the care recipients’ education level and grade of disability.

The DID specification is the same as equation (1). In the DID approach, we had two observations for 25 651 persons with heavy disabilities, one before they signed the agreement (insurance = 0) and the other when they signed the agreement (insurance =1). We also included 523 persons, who were assessed as with moderate disabilities initially and had applied at least twice by September 2020 and with assessment scores being either 45 or 50 at their first application. Among these 523 persons, 473 were assessed as having heavy disabilities after their second application. Therefore, these 473 persons were not treated (insurance = 0) until their third observation when they signed the agreement. For the remaining 50 of these 523 persons, we did not have their third observation because they either were still rejected after their second application or had not signed the agreement by the end of our observation window. That is, they were never treated. See supplementary Table S3 (see online supplemtary material) for the data structure. For robustness, we compared the results when controlling for marital status, disability grade, mental health conditions, and conditions that demanded LTC with those when not.

Those with moderate disabilities were ineligible for the benefit, but the policy allowed them to apply again 6 months later. Every time they applied, they filled an application form and reported their then living conditions and residential address. Seeking LTCI at least twice suggests that they had some similarity to those with heavy disabilities.

In an alternative regression, we restricted the treated observations to those older persons with heavy disabilities after their application and with assessment scores being either 40 or 35. This strategy was now similar to a regression discontinuity design in that we only included those observations nearly above the cutoff point, 40 here, as the control observations, and those nearly below as the treated ones. This restriction makes the control and the treated observations closer to each other.

Under the previous reasoning that there was no systematic time trend shifting the insured’s choice of place of ageing, the event study and the DID approach should deliver identical estimates. For more details on the comparison between DID and event study, see supplementary Note 2 in the supplementary online material.

We adopted a linear probability model (LPM) for both event study and DID approaches. Although the dependent variable is binary in our case, the LPM does a good job in estimating the average effects of explanatory variables (Wooldridge 2010). Standard errors were robust ones in both approaches.

## Results

### Sample characteristics

Before LTCI, home was the major choice by those with heavy disabilities (Table 1). At the time of application, 18 741 (71.7%) and 7383 (28.3%) persons chose home and institution respectively. Among those with heavy disabilities, females accounted for 56%, nearly 90% of them were >65 years old, about half (53.7%) of them had primary or secondary school education, while 23.8% of them were illiterate. Regarding the duration of disability by application, 35.5% had been diagnosed with disabilities for ≤1 year, and 18.6% for >5 years. Regarding disability grade, 63.2 and 35.9% of them were classified as with grade-one and grade-two heavy disabilities, respectively. Those with grade-three heavy disabilities only accounted for 0.9%. Most had some conditions that demanded LTC. In addition, 18.8% of them were also diagnosed with some mental health conditions.

**Table 1.** Characteristics of the insured by place of ageing before they signed the LTCI agreement; all applicants in City X between July 2017 and September 2020

	Total (*n* = 26 124)		Home (*n* = 18 741)		Institution (*n* = 7383)	
	*n*	%	*n*	%	*n*	%
Sex						
Male	11 488	43.97	8471	45.20	3017	40.86
Female	14 636	56.03	10 270	54.80	4366	59.14
Age, years						
≤65	2870	10.99	2347	12.52	523	7.08
66–75	5164	19.77	4031	21.51	1133	15.35
76– 85	9894	37.87	6790	36.23	3104	42.04
86– 95	7336	28.08	4944	26.38	2392	32.40
>95	860	3.29	629	3.36	231	3.13
Marriage						
No spouse	12 161	46.55	7918	42.25	4243	57.47
Having spouse	13 963	53.45	10 823	57.75	3140	42.53
Education						
Illiterate	6208	23.76	4879	26.03	1329	18.00
Primary and secondary school	14 035	53.72	10 261	54.75	3774	51.12
High school and above	5881	22.51	3601	19.21	2280	30.88
Months of disability by application						
≤12	9261	35.45	6196	33.06	3065	41.51
13–24	5160	19.75	3707	19.78	1453	19.68
25–36	3289	12.59	2387	12.74	902	12.22
37–48	1896	7.26	1418	7.57	478	6.47
49–60	1657	6.34	1229	6.56	428	5.80
>60	4861	18.61		20.30	1057	14.32
Disability grade						
Grade-one heavy	16 515	63.22	12 803	68.32	3712	50.28
Grade-two heavy	9384	35.92	5816	31.03	3568	48.33
Grade-three heavy	225	0.86	122	0.65	103	1.40
Mental health conditions						
No	18 407	70.46	13 592	72.53	4815	65.22
Yes	4918	18.83	3177	16.95	1741	23.58
Missing	2799	10.71	1972	10.52	827	11.20
Conditions that demand LTC^a^						
No	8742	33.46	6392	34.11	2350	31.83
Yes	17 382	66.54	12 349	65.89	5033	68.17

a In their application forms, applicants needed to tell whether they had been diagnosed with any of nearly 30 diseases; among these reported diseases, Parkinson’s disease, fracture, cerebral infarction, and stroke were classified as conditions that demanded LTC.

Comparison between those who chose home with those who chose institution shows that the latter were older, less likely to have a living spouse, and more educated. In addition to the difference in demographics, these two groups also differed in health status. The latter were less likely to have been diagnosed with disability for >5 years by the time of application, more likely to have grade-two or grade-three heavy disabilities, and more likely to have mental health conditions (Table 1).

### Impact of becoming eligible for LTCI

Direct tabulation shows that becoming eligible for the LTCI benefit moved a large portion of those in institutions, either in nursing hospitals or in older-adult care institutions, to homes (Table 2). Out of the 3619 (3764) individuals who lived in nursing hospitals (older-adult care institutions) before, 61.6% (55.1%) moved to homes once becoming eligible; almost all of the 18 741 individuals who lived in homes before still chose to live in homes after becoming eligible.

**Table 2.** Choice of place of ageing^a^ before and when the insured signed the agreement; all applicants in City X between July 2017 and September 2020

	Post-LTCI setting		
Original setting	Home	Institution	Total
Home	18 658 (99.6%)	83 (0.4%)	18 741 (100%)
Nursing hospital	2229 (61.6%)	1390 (38.4%)	3619 (100%)
Older-adult care institution	2074 (55.1%)	1690 (44.9%)	3764 (100%)
Total	22 961	3163	26 124

a The initial choice of place of ageing under original setting was inferred from the application data based on living conditions and residential address reported by applicants. Those living in nursing hospitals or older-people care institutions were classified as living in institutions and all the others as living in homes. The choice of place of ageing after one becoming eligible for but before starting to receive any actual benefit under post-LTCI setting was inferred from the agreement data directly based on care type (home or institutional care) chosen by care recipients.

The event study delivered estimates in the same direction (Table 3). The first column shows that after becoming eligible, the choice of home as the place of ageing increased by 16.4%, statistically significant at the 1% confidence level. Restricting the sample to those who applied before July 2020 in the second column barely changed the result. Restricting the sample only to those who applied after July 2020 in the third column changed the result, but only slightly. In the fourth column we controlled for the binary variable denoting the policy adjustment after July 2020 and its interaction with the independent variable of interest. As shown and also as expected, the positive impact on the insured’s willingness to shift to homes was weakened after the adjustment, but only slightly—the estimated main effect was 16.5% and the interaction effect was only −2.5%—the choice of home still increased by 14.0% net. Dropping those persons with time intervals between applying for the benefit and signing the agreement of >100 days hardly affected the estimates (supplementary Table S4, see online supplementary material).

**Table 3.** Event-study estimates^a^ of the impact of becoming eligible for the LTCI benefit on choice of place of ageing; all applicants in City X between July 2017 and September 2020

	(1)	(2)	(3)	(4)
	Whole sample	Before July 2020	After July 2020	Policy adjustment
Insurance	0.164*** (0.003)	0.165*** (0.003)	0.157*** (0.014)	0.165*** (0.003)
Insurance post July 2020				−0.025** (0.012)
Months of disability by application (ref: ≤12)				
13–24	0.001 (0.009)	0.003 (0.009)	−0.035 (0.035)	0.001 (0.009)
25–36	−0.002 (0.013)	0.002 (0.013)	−0.109* (0.058)	−0.002 (0.013)
37–48	−0.005 (0.017)	0.001 (0.017)	−0.214*** (0.076)	−0.005 (0.017)
49–60	−0.018 (0.022)	−0.011 (0.022)	−0.371*** (0.084)	−0.019 (0.022)
>60	−0.011 (0.026)	−0.002 (0.027)	−0.475*** (0.108)	−0.013 (0.026)
Year (ref: 2017)				
2018	−0.035*** (0.010)	−0.036*** (0.010)		−0.036*** (0.010)
2019	−0.081*** (0.020)	−0.084*** (0.020)		−0.083*** (0.020)
2020	−0.090*** (0.032)	−0.093*** (0.032)		−0.093*** (0.032)
Constant	0.755*** (0.012)	0.750*** (0.012)	0.772*** (0.019)	0.756*** (0.012)
Individual fixed effects	Yes	Yes	Yes	Yes
Observations	52 248	50 396	1852	52 248
Number of persons	26 124	25 198	926	26 124
*R* ^2^	0.156	0.157	0.140	0.156

a Robust standard errors in parentheses.

*** *P* < 0.01,

** *P* < 0.05,

* *P* < 0.1.

We report our DID results in Table 4. The estimation sample includes all 26 174 persons introduced in supplementary Table S3. Supplementary Table S5 (see online supplementary material) contains summary statistics of those 523 persons introduced in the second and the third column in Supplementary Table S3 at their first application. The first two columns in Table 4 show that the choice of home as the place of ageing increased by 16.4%, identical with that of the event study. This confirms our previous reasoning that there was no systematic time trend shifting the insured’s choice of place of ageing between their applying for the benefit and signing the agreement.

**Table 4.** DID estimates^a^ of the impact of becoming eligible for the LTCI benefit on choice of place of ageing

	All with heavy disabilities		Those with heavy disabilities and with scores of 35 or 40	
	(1)	(2)	(3)	(4)
Insurance	0.164*** (0.003)	0.164*** (0.003)	0.086*** (0.005)	0.083*** (0.005)
Months of disability by application (ref: ≤ 12)				
13–24	0.005 (0.008)	0.003 (0.008)	0.012 (0.014)	0.007 (0.014)
25–36	0.003 (0.012)	0.001 (0.012)	0.015 (0.019)	0.008 (0.019)
37–48	0.006 (0.015)	0.002 (0.015)	0.037 (0.024)	0.030 (0.024)
49–60	−0.001 (0.018)	−0.006 (0.018)	0.013 (0.028)	0.003 (0.028)
>60	0.009 (0.021)	0.003 (0.021)	0.011 (0.030)	−0.001 (0.030)
Year (ref: 2017)				
2018	−0.052*** (0.009)	−0.034*** (0.010)	−0.069*** (0.015)	−0.030* (0.017)
2019	−0.122*** (0.015)	−0.077*** (0.018)	−0.138*** (0.023)	−0.058* (0.031)
2020	−0.154*** (0.025)	−0.087*** (0.029)	−0.167*** (0.038)	−0.037 (0.055)
Marriage		0.068 (0.050)		0.065 (0.047)
Disability grade (ref: grade-one heavy)				
Mild		−0.029 (0.140)		−0.060 (0.132)
Grade-one moderate		0.052*** (0.019)		0.041 (0.025)
Grade-two moderate		0.083* (0.047)		0.043 (0.054)
Grade-two heavy		−0.048 (0.034)		−0.076** (0.035)
Grade-three heavy		−0.255 (0.231)		−0.520** (0.224)
Mental health conditions (ref: no)				
Yes		0.030 (0.030)		0.032 (0.036)
Missing		−0.004 (0.042)		−0.019 (0.052)
Conditions that demand LTC^b^		−0.002 (0.027)		−0.018 (0.031)
Constant	0.768*** (0.010)	0.729*** (0.039)	0.910*** (0.017)	0.855*** (0.042)
Individual fixed effects	Yes	Yes	Yes	Yes
Observations	52 821	52 821	8531	8531
Number of persons	26 174	26 174	4233	4233
*R* ^2^	0.153	0.154	0.079	0.087

a Robust standard errors in parentheses.

b In their application forms, applicants needed to tell whether they had been diagnosed with any of nearly 30 diseases. Among these reported diseases, Parkinson’s disease, fracture, cerebral infarction, and stroke were classified as conditions that demanded LTC. Since additional controls in column (2) and (4)—marital status, disability grade, mental health conditions, and conditions that demanded LTC—were reported in the application data rather than in the agreement data, we had to assume that as control variables, they incurred no change between the insured last applying for the benefit and signing the agreement. Given that the time intervals between these two time points were short for most insured (supplementary Fig. S2), this assumption is reasonable.

*** *P* < 0.01,

** *P* < 0.05,

* *P* < 0.1.

Restricting the treated observations to those with heavy disabilities and with scores being either 40 or 35 in the third (fourth) column reduced the estimate to 8.6 (8.3)%. This reduction was consistent with our subsequent heterogeneity analysis shown in Table 5, where the treatment effect was larger for those with heavier disabilities.

**Table 5.** Heterogeneity analysis of the impact of becoming eligible for the LTCI benefit on choice of place of ageing; all applicants in City X between July 2017 and September 2020

	(1)	(2)
Variables	Education	Grade of disability
Insurance	0.124*** (0.005)^a^	0.134*** (0.003)
Insurance × primary and secondary school	0.032*** (0.005)	
Insurance × high school and above	0.103*** (0.007)	
Insurance × grade-two heavy		0.083*** (0.005)
Insurance × grade-three heavy		0.099*** (0.029)
Months of disability by application (ref: ≤12)		
13–24	0.000 (0.009)	0.002 (0.009)
25–36	−0.002 (0.013)	−0.005 (0.013)
37–48	−0.006 (0.017)	−0.013 (0.017)
49–60	−0.020 (0.022)	−0.029 (0.022)
>60	−0.012 (0.026)	−0.029 (0.026)
Year (ref: 2017)		
2018	−0.034*** (0.010)	−0.034*** (0.010)
2019	−0.081*** (0.020)	−0.076*** (0.020)
2020	−0.086*** (0.032)	−0.080** (0.032)
Constant	0.755*** (0.012)	0.757*** (0.012)
Individual fixed effects	YES	YES
Observations	52 248	52 248
Number of persons	26 124	26 124
*R* ^2^	0.164	0.166

a Robust standard errors in parentheses.

*** *P* < 0.01,

** *P* < 0.05.

Finally, we analyzed the heterogeneity (Table 5). The first column shows that compared with the illiterate, the impact of the eligibility for those with primary or secondary school education (high school education or above) was larger by 3.2 (10.3)%. The second column shows that compared with those with grade-one heavy disabilities, the impact for those with grade-two (grade-three) heavy disabilities was larger by 8.3 (9.9)%. This heterogeneity was robust when we applied the same analysis to the DID sample (supplementary Table S6, see online supplementary material).

### Parallel trends

To validate the assumption of parallel trends required for the DID approach, we followed Miller (2023) and estimated the following model,


(2) $$\begin{array}{*{20}{c}}
Home\_car{e_{it}} &= \,{{\Sigma }_{{\mathrm{j}} \in \left\{ { - 12, \ldots ,0, \ldots ,12} \right\}}}{\gamma _j}\, \cdot Insuranc{e_{it - j}} \\ & \quad + \beta \cdot Contro{l_{it}} + {\alpha _i} + Yea{r_t} + {\varepsilon _{it}},
\end{array}$$

where $t$ represents the calendar month for each observation; $j$ represents event month, or the month when the insured signed the agreement in our context; $t - j$ means that the insured signed the agreement with local agencies $j$ months before (if $j > 0$) or after (if $j < 0$) the current calendar month.

This model estimates the dynamic effect of becoming eligible for LTCI on the choice of place of ageing. If the coefficients ${{\gamma }_{\mathrm{j}}}\,(j < 0)$ are not significantly different from zero, the assumption of parallel trends is likely to hold. All other covariates are the same as those in equation (1).

Our data was limited in the sense that we did not observe the whole pre-treatment history for the insured: we could only observe their place of ageing at one or two time points before they signed the agreement. Nevertheless, the months between the insured applying for the benefit and signing the agreement varied across individuals, as shown in supplementary Fig. S2. This feature enabled us to estimate pre-treatment dynamics.

The agreement dataset also recorded any change of place of ageing after the insured signed the agreement, which enabled us to construct the post-treatment history of place of ageing for them.

**Figure 1.**
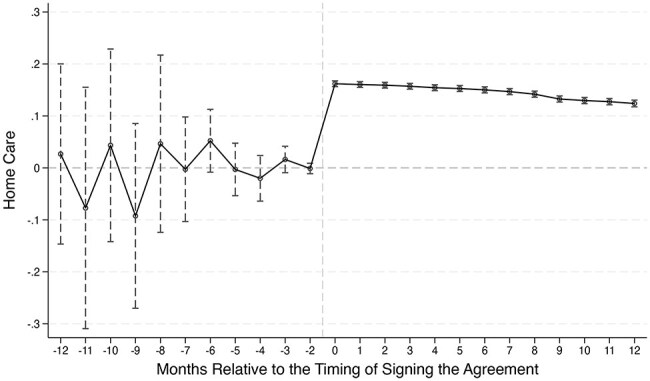
Parallel trends. Estimation of the dynamic effect of becoming eligible for LTCI on the insured’s choice of place of ageing shows no significant pre-trend.

Figure 1 shows the estimates of the dynamic effect ${\gamma _j}$. All coefficients fluctuate around zero and are in fact not significantly different from zero before the insured signed the agreement. This validates our previous reasoning that there was no systematic time trend shifting the insured’s choice of place of ageing between their applying for the benefit and signing the agreement.

Once the insured become eligible for the LTCI benefit, the coefficient jumps to ∼0.16, and remains around 0.12 even 12 months afterwards, showing a large and persistent policy effect of the LTCI eligibility on the insured’s choice of place of ageing.

## Discussion

Our results show that becoming eligible for the LTCI in City X caused more insured to choose home as their place of ageing and this positive impact was larger for those of higher education level or of higher disability grade.

The results are consistent with our hypothesis that becoming eligible for the LTCI in City X caused more insured to choose home as their place of ageing because the LTCI could directly compensate family caregivers’ opportunity cost.

More shift to homes from institutions by those of higher education level or of higher disability grade could both arise from the possibility that their family caregivers, previously without the LTCI, had to incur higher opportunity cost in the first place. First, the bigger shift to homes by those of higher education level could result from the fact that their family caregivers also had higher education partly because of assortative matching (supplementary Table S7, see online supplementary material). Higher education level means higher labor income outside and thus higher opportunity cost of providing family care. This caused more institutional care in the first place, consistent with the data pattern (supplementary Table S8, see online supplementary material).

Second, those of higher disability grade generally required more intensive care. This meant that their family caregivers had to sacrifice more and thus bear higher opportunity cost. Therefore, before the introduction of LTCI, home was less likely to be their first choice, also consistent with the data pattern (supplementary Table S8).

Our study has a number of strengths. First, our administrative dataset contains accurate records on all program applicants from a pilot city in China. This lends our quantitative estimates a high degree of statistical power. Second, in our DID approach, we constructed the control observations based on applicants’ assessment scores and thus made them quite comparable to the treated ones.

Our study contributes to the domestic and international literature on the design of LTCI. While most domestic studies on China’s LTCI focused on its impact on hospitalization and medical expenditure (Feng et al. 2020a, Lu et al. 2020, Chen and Ning 2022, Liu et al. 2023b), we believe that it is also a priority to align the design of LTCI with older people’s preference for ageing in place in order to promote healthy ageing.

In the international literature, most studies on LTCI have drawn evidence from developed countries, including the USA (Van Houtven and Norton 2004, McKnight 2006), Canada (Stabile et al. 2006), Europe (Bonsang 2009), and Korea (Kim and Lim 2015). Our study provides evidence from a developing country. China is not only the largest developing country with the largest number of older people, but also distinct in its Confucianism. These all make its experience valuable. As far as we know, the LTCI in City X is most similar to that in Germany which also emphasizes home-based informal care (Gerlinger 2018). Our result indicates that LTCI would help more people achieve ageing in place if informal care is compensated alongside formal institutional care. Moreover, we took the introduction of a completely new LTCI system as the study context, while most previous international studies were based on existing systems (Van Houtven and Norton 2004, McKnight 2006, Guo et al. 2015).

Our research also speaks to the growing literature on LTCI’s impact on medical expenditure. An increased shift to homes from institutions and the fact that institutions defined here include both nursing hospitals and older-adult care institutions implies that the medical expenditure of the insured at hospitals was likely to fall. This is consistent with previous findings that the introduction of LTCI in China reduced hospitalization and medical expenditure (Feng et al. 2020a, Lu et al. 2020, Chen and Ning 2022). These findings also suggested the substitution of LTC for hospital care as a cause for the reduction. Our study instead presents direct evidence of the substitution of home care for institutional care, which includes hospital care in our context, and thus shows that LTCI could reduce medical expenditure by decreasing potential bed-blocking. In the settings of previous research, only formal care was subsidized, and researchers attributed the decline in medical expenditure to the substitution of older-adult care institutions for hospitals. However, our study reveals that medical expenditure could also decrease when LTCI compensated for both home and institutional care and the insured substituted the former for the latter once becoming eligible for the benefit.

Finally, an increased shift to homes implies that family caregivers’ labor force participation and labor supply to the outside market were likely to fall as a result, and this finding contributes to the literature exploring the impact of LTCI on labor force participation and labor supply of family caregivers (Geyer and Korfhage 2015, Fu et al. 2017). The change in family members’ labor supply implies that LTCI would have a broader impact on the economy, especially when the population of the insured is large.

### Limitations

Our study had some limitations. First, COVID-19 caused a sudden drop of applications between February 2020 and June 2020. Moreover, the lock-down policy in early 2020 in China might have affected the availability of institutional care then, which might affect the interpretation of our results. Second, our quantitative estimates are highly specific to the group of people in the UEBMI scheme with heavy disabilities in City X, as each pilot program in China has its own design and features (supplementary Table S1). Therefore, one should be careful in extrapolating our results to other cities or countries.

## Conclusion

The design of the LTCI in City X, by providing benefit not only to institutions, but also to family caregivers, moderated the conflict between traditional filial piety and the modern market-based economy, helped more eligible older people achieve ageing in place, and thus promoted healthy ageing. Healthy ageing is more than freedom from disease. Subjective well-being also plays an important role (Chen et al. 2022). Ageing in place, aligned well with older people’s preference, directly contributes to their subjective well-being.

Note that in the first round of the LTCI pilot in China, 3 cities out of 15 in total provided cash transfers to informal care (supplementary Table S1). In the second round of the LTCI pilot starting from late 2020, among the 14 new pilot cities that provided LTCI policy details, 8 chose to cover informal home care. This indicates that informal home care got more acknowledged and emphasized in policy making. Our study provides lessons not only for those pilot cities that already covered both home and institutional care, but also for other cities with home care on their agenda. Covering informal home care could encourage ageing in place. This is aligned well with older people’s preference, improves their subjective well-being, and thus promotes healthy ageing. Those areas that lack institutional care or with an underdeveloped market for formal care would benefit from covering informal home care as well. In addition, covering informal home care could also reduce medical expenditure as the insured substitute home care for hospital care and decrease potential bed-blocking. Those areas with tight public finances for medical insurance and a limited number of hospital beds for inpatients would also benefit from including this option.

Ageing necessitates the introduction of LTCI for many developing countries. Our research offers rigorous quantitative evidence from a pilot city in China and could provide lessons for other developing countries, particularly for East Asian countries that share similar Confucian culture. Our study suggests that the specifics of the LTCI design, such as who could receive subsidies, family values, and family members’ engagement in the outside labor market, together impact older people’s choice of place of ageing.

## Supplementary Material

czae109_Supp

## Data Availability

The data underlying this article could not be shared publicly as it involves the privacy of those individuals in the administrative data.

## Reflexivity statement

Z.C. is a female assistant professor based in Southwest China; H.Z. is a female working in a local government tax bureau based in North China; X.M. is a male assistant professor based in Southwest China. Z.C. and H.Z. have expertise in health policy and ageing, and X.M. has expertise in economics and applied econometrics.
