# Supplementary material for: Becoming eligible for long-term care insurance in China brought more ageing at home: evidence from a pilot city
Source: Health Policy Plan. 2024 Nov 9;40(2):165–75. doi: 10.1093/heapol/czae109 (PMC11884802; doi:10.1093/heapol/czae109)
Supplement: czae109_Supp [file czae109_supp.zip › New folder/Supplementary Information_Final.docx]

**
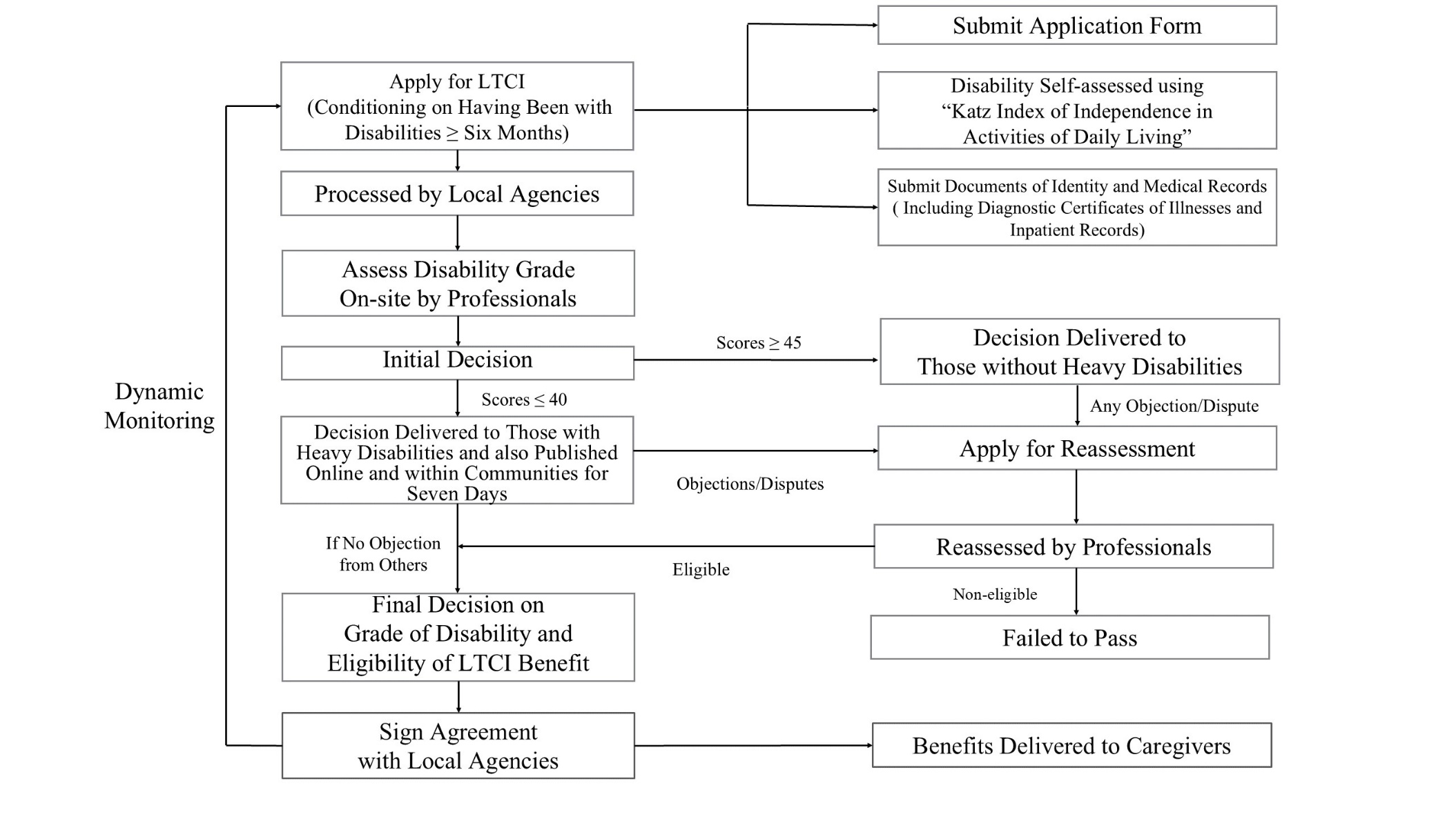
**

**Supplementary Figure 1. LTCI Application Process in City X**


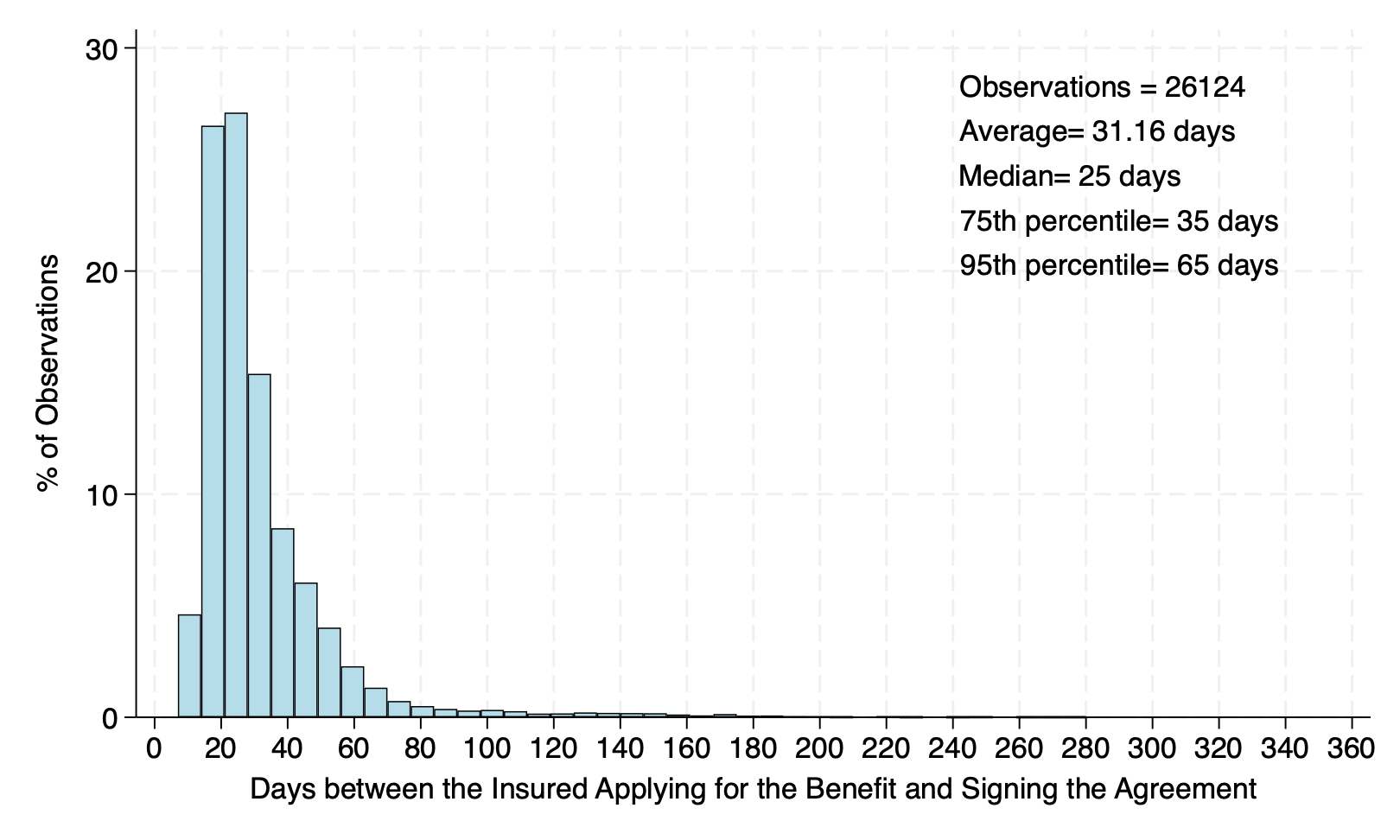


**Supplementary Figure 2. Distribution of the Time Intervals between the Insured Applying for the Benefit and Signing the Agreement, All Applicants in City X between Jul. 2017 and Sep. 2020**

*Note:* The distribution is truncated at 360 days.

**Supplementary Table 1. Summary of the LTCI Programs in 15 Pilot Cities**

| **Types of Care Covered** | **Pilot City**  **(Starting Time)** | **Percentage of Expenses Reimbursed (Maximum Quota)**  **or Benefit Amount** |
| --- | --- | --- |
| Institutional Care (IC);  Formal & Informal Home Care (HC); | Chengdu  (Jul. 2017) | A fixed benefit amount, differed by HC or IC and the assessed grade of disability. |
|  | Shihezi  (Jan. 2017) | 70% (≤750 CNY per month) ^a^ for IC;  25 CNY per day for formal and informal HC. |
|  | Shangrao  (Jan. 2017) | 1,080 CNY per month for IC;  900 CNY per month for formal HC;  100% (≤180 CNY per month) for renting care facilities in formal HC;  450 CNY per month for informal HC. |
| IC;  Formal HC | Qingdao  (Apr. 2018) | 90% for employees insured;  80% or 70% for residents insured. |
|  | Tsitsihar  (Oct. 2017) | 60% (≤30 CNY per day) for nursing hospitals;  55% (≤25 CNY per day) for nursing homes;  50% (≤10 CNY per day) for formal HC. |
|  | Chongqing  (Dec. 2018) | 50 CNY per day. |
|  | Chengde  (Jun. 2017) | 70% (≤60 CNY per day) for nursing hospitals;  70% (≤50 CNY per day) for nursing homes;  70% (≤40 CNY per day) for formal HC. |
|  | Nantong  (Jan. 2016) | 60% for nursing hospitals;  50% for nursing homes;  100% (≤1,200 CNY per month) for formal HC. |
|  | Anqing  (Jan. 2017) | 60% (≤50 CNY per day) for nursing hospitals;  50% (≤40 CNY per day) for nursing homes;  100% (≤750 CNY per month) for formal home care provided by designated agencies;  15 CNY per day for formal home care provided by non-designated agencies. |
|  | Guangzhou  (Aug. 2017) | 75% (≤120 CNY per day) for IC;  90% (≤115 CNY per day) for formal HC;  100% (≤1,000 CNY per month) for medical care. |
|  | Suzhou  (Oct. 2017) | IC:  26 CNY per day for heavy disabilities;  20 CNY per day for moderate disabilities.  Formal HC:  30 CNY per day for heavy disabilities;  25 CNY per day for moderate disabilities. |
|  | Shanghai  (Jan. 2017) | 85% for IC;  90% for formal HC. |
|  | Jingmen  (Jan. 2017) | 70% (≤150 CNY per day) for nursing hospitals;  75% (≤100 CNY per day) for nursing homes;  80% (≤100 CNY per day) for full-time formal HC;  80% (≤40 CNY per day) for part-time formal HC. |
| IC | Ningbo  (Dec. 2017) | 40 CNY per day. |
|  | Changchun  (May. 2015) | 90% for employees insured;  80% for residents insured. |

*Source:* Authors collected from various government websites and newspapers.

*Note:* ^a^ Those in parentheses are quotas set by each local government.

**Supplementary Table 2. LTCI Benefit in Jul. 2020 in City X (CNY Per Month)**

| **Disability Grade** | **Home Care** | | **Institutional Care** |
| --- | --- | --- | --- |
|  | **Informal** | **Formal Home** |  |
| Grade-One Heavy | 1,077 | 300 | 1,377 |
| Grade-Two Heavy | 1,437 | 400 | 1,837 |
| Grade-Three Heavy | 1,796 | 500 | 2,296 |

*Source:* Authors collected from City X’s government website. Those who participated in the professional training could get the full benefit for family caregivers; otherwise, they could only get 80% of the full benefit, that is, 862, 1150, 1437 CNY per month for the insured with grade-one, grade-two, and grade-three heavy disabilities, respectively.

**Supplementary Table 3. Data Structure in DID Analysis**

|  | Value of *Insurance* | | |
| --- | --- | --- | --- |
| First Application | 0 | 0 | 0 |
| Second Application |  | 0 | 0 |
| Signing Agreement | 1 | 1 |  |
| Number of Persons | 25,651 | 473 | 50 |

*Note:* The 26,174 persons here constituted the sample in the DID analysis. Our event study only involved before and after comparison, and the sample there included both observations of the persons in the first column and the last two observations (out of three observations in total) of the persons in the second column. We only kept their last two application records for the 523 persons in the second and the third column. Out of the 50 persons in the third column, 42 of them were still rejected after their second application, and the remaining eight were classified as with heavy disabilities after their second application. Unfortunately, these eight persons either passed away before they signed the agreement or had not yet signed the agreement by the end of our observation window. Therefore, we only had two observations for these eight persons.

**Supplementary Table 4. Robustness Check by Excluding Those with Time Intervals between They Applying for the Benefit and Signing the Agreement Greater than 100 Days, All Applicants in City X between Jul. 2017 and Sep. 2020**

|  | (1) | (2) | (3) | (4) |
| --- | --- | --- | --- | --- |
|  | Whole Sample | Before July 2020 | After July 2020 | Policy Adjustment |
| Insurance | 0.165*** | 0.165*** | 0.157*** | 0.166*** |
|  | (0.003) | (0.003) | (0.014) | (0.003) |
| Insurance*Post July 2020 |  |  |  | -0.025** |
|  |  |  |  | (0.012) |
| Months of Disability by Application (Ref:<=12) |  |  |  |  |
| 13~24 | -0.000 | 0.002 | -0.035 | 0.000 |
|  | (0.009) | (0.009) | (0.035) | (0.009) |
| 25~36 | -0.003 | 0.001 | -0.109* | -0.003 |
|  | (0.013) | (0.014) | (0.058) | (0.013) |
| 37~48 | -0.005 | 0.001 | -0.214*** | -0.005 |
|  | (0.017) | (0.018) | (0.076) | (0.017) |
| 49~60 | -0.018 | -0.010 | -0.371*** | -0.019 |
|  | (0.022) | (0.023) | (0.084) | (0.022) |
| >60 | -0.010 | -0.001 | -0.475*** | -0.012 |
|  | (0.027) | (0.027) | (0.108) | (0.027) |
| Year (Ref: 2017) |  |  |  |  |
| 2018 | -0.036*** | -0.037*** |  | -0.037*** |
|  | (0.010) | (0.010) |  | (0.010) |
| 2019 | -0.084*** | -0.087*** |  | -0.086*** |
|  | (0.020) | (0.020) |  | (0.020) |
| 2020 | -0.091*** | -0.094*** |  | -0.094*** |
|  | (0.032) | (0.032) |  | (0.032) |
| Constant | 0.756*** | 0.751*** | 0.772*** | 0.757*** |
|  | (0.012) | (0.012) | (0.019) | (0.012) |
| Individual FE | YES | YES | YES | YES |
| Observations | 51,096 | 49,244 | 1,852 | 51,096 |
| Number of IDs | 25,548 | 24,622 | 926 | 25,548 |
| R-squared | 0.156 | 0.157 | 0.140 | 0.156 |

*Note:* In the data, there were 576 persons with time intervals between they applying for the benefit and signing the agreement greater than 100 days. Most of these applications (N=549) happened in year 2020: all of them applied before June 2020, but signed the agreement during the latter half of the year. Probably Covid-19 caused the postponement. Robust standard errors in parentheses. *** p<0.01. ** p<0.05. * p<0.1.

**Supplementary Table 5. Characteristics of Those with Moderate Disabilities by Types of Care, All Applicants in City X between Jul. 2017 and Sep. 2020**

|  | Total  (N= 523) | | Home  (N= 449) | | Institution  (N = 74) | |
| --- | --- | --- | --- | --- | --- | --- |
|  | N | % | N | % | N | % |
| Sex |  |  |  |  |  |  |
| Male | 242 | 46.27 | 211 | 46.99 | 31 | 41.89 |
| Female | 281 | 53.73 | 238 | 53.01 | 43 | 58.11 |
| Age |  |  |  |  |  |  |
| <=65 | 59 | 11.28 | 55 | 12.25 | 4 | 5.41 |
| 66~75 | 157 | 30.02 | 142 | 31.63 | 15 | 20.27 |
| 76~85 | 185 | 35.37 | 152 | 33.85 | 33 | 44.59 |
| 86~95 | 104 | 19.89 | 83 | 18.49 | 21 | 28.38 |
| >95 | 18 | 3.44 | 17 | 3.79 | 1 | 1.35 |
| Marriage |  |  |  |  |  |  |
| No Spouse | 203 | 38.81 | 160 | 35.63 | 43 | 58.11 |
| Having Spouse | 320 | 61.19 | 289 | 64.37 | 31 | 41.89 |
| Education |  |  |  |  |  |  |
| Illiterate | 126 | 24.09 | 111 | 24.72 | 15 | 20.27 |
| Primary and Secondary School | 293 | 56.02 | 257 | 57.24 | 36 | 48.65 |
| High School and Above | 104 | 19.89 | 81 | 18.04 | 23 | 31.08 |
| Months of Disability by Application | |  |  |  |  |  |
| <=12 | 114 | 21.8 | 92 | 20.49 | 22 | 29.73 |
| 13~24 | 120 | 22.94 | 108 | 24.05 | 12 | 16.22 |
| 25~36 | 77 | 14.72 | 63 | 14.03 | 14 | 18.92 |
| 37~48 | 35 | 6.69 | 31 | 6.9 | 4 | 5.41 |
| 49~60 | 47 | 8.99 | 40 | 8.91 | 7 | 9.46 |
| >60 | 130 | 24.86 | 115 | 25.61 | 15 | 20.27 |
| Disability Grade ^a^ | | |  |  |  |  |
| Grade-One Moderate | 468 | 89.48 | 400 | 89.09 | 68 | 91.89 |
| Grade-Two Moderate | 55 | 10.52 | 49 | 10.91 | 6 | 8.11 |
| Mental Health Conditions |  |  |  |  |  |  |
| No | 412 | 78.78 | 357 | 79.51 | 55 | 74.32 |
| Yes | 105 | 20.08 | 86 | 19.15 | 19 | 25.68 |
| Missing | 6 | 1.15 | 6 | 1.34 | 0 | 0 |
| Conditions that Demand LTC ^b^ | | |  |  |  |  |
| No | 149 | 28.49 | 128 | 28.51 | 21 | 28.38 |
| Yes | 374 | 71.51 | 321 | 71.49 | 53 | 71.62 |

*Note:* ^a^ The disability grade for those with moderate disabilities refers to the grade assessed at their first application. Similarly, all the other information refers to those filled at their first application. ^b^ In their application forms, applicants needed to tell whether they had been diagnosed with any of nearly 30 diseases. Among these reported diseases, Parkinson's disease, fracture, cerebral infarction, and stroke were classified as conditions that demanded LTC.

**Supplementary Table 6. Heterogeneity Analysis of the Impact of Becoming Eligible for the LTCI Benefit on Choices of Places of Ageing Using DID Sample**

|  | (1) | (2) | (3) | | (4) |
| --- | --- | --- | --- | --- | --- |
|  | Education | | Grade of Disability | | |
| Insurance | 0.124*** | 0.123*** | 0.134*** | | 0.133*** |
|  | (0.004) | (0.004) | (0.003) | | (0.003) |
| Insurance × Primary and | 0.032*** | 0.032*** |  | |  |
| Secondary School | (0.005) | (0.005) |  | |  |
| Insurance × High School | 0.103*** | 0.103*** |  | |  |
| and above | (0.007) | (0.007) |  | |  |
| Insurance × Grade-Two |  |  | 0.083*** | | 0.083*** |
| Heavy |  |  | (0.005) | | (0.005) |
| Insurance × Grade-Three |  |  | 0.099*** | | 0.099*** |
| Heavy |  |  | (0.029) | | (0.029) |
| Months of Disability by Application (Ref: <=12) | | |  | |  |
| 13~24 | 0.004 | 0.003 | 0.005 | | 0.005 |
|  | (0.008) | (0.008) | (0.008) | | (0.008) |
| 25~36 | 0.003 | 0.000 | -0.001 | | -0.001 |
|  | (0.012) | (0.012) | (0.012) | | (0.012) |
| 37~48 | 0.005 | 0.002 | -0.004 | | -0.003 |
|  | (0.015) | (0.015) | (0.015) | | (0.015) |
| 49~60 | -0.002 | -0.007 | -0.013 | | -0.012 |
|  | (0.018) | (0.018) | (0.018) | | (0.018) |
| >60 | 0.008 | 0.001 | -0.010 | | -0.009 |
|  | (0.021) | (0.021) | (0.021) | | (0.021) |
| Year (Ref: 2017) |  |  |  | |  |
| 2018 | -0.051*** | -0.034*** | -0.033*** | | -0.033*** |
|  | (0.009) | (0.010) | (0.010) | | (0.010) |
| 2019 | -0.122*** | -0.076*** | -0.074*** | | -0.073*** |
|  | (0.015) | (0.018) | (0.018) | | (0.018) |
| 2020 | -0.150*** | -0.083*** | -0.083*** | | -0.078*** |
|  | (0.025) | (0.029) | (0.028) | | (0.029) |
| Marriage |  | 0.071 |  | | 0.069 |
|  |  | (0.050) |  | | (0.050) |
| Disability Grade (Ref: Grade-One Heavy) | | | |  | |
| Mild |  | -0.031 | -0.052 | | -0.046 |
|  |  | (0.139) | (0.143) | | (0.141) |
| Grade-One Moderate |  | 0.053*** | 0.043** | | 0.038** |
|  |  | (0.019) | (0.018) | | (0.019) |
| Grade-Two Moderate |  | 0.078* | 0.065 | | 0.068 |
|  |  | (0.047) | (0.047) | | (0.047) |
| Grade- Two Heavy |  | -0.047 | -0.090*** | | -0.089*** |
|  |  | (0.034) | (0.034) | | (0.034) |
| Grade- Three Heavy |  | -0.252 | -0.318 | | -0.306 |
|  |  | (0.231) | (0.238) | | (0.230) |
| Mental Health Conditions (Ref: No)  Yes | | 0.030 |  | | 0.029 |
|  |  | (0.030) |  | | (0.030) |
| Missing |  | -0.005 |  | | -0.010 |
|  |  | (0.041) |  | | (0.042) |
| Conditions that Demand LTC | | -0.003 |  | | -0.003 |
|  |  | (0.027) |  | | (0.027) |
| Constant | 0.767*** | 0.727*** | 0.786*** | | 0.746*** |
|  | (0.010) | (0.039) | (0.017) | | (0.039) |
| Individual FE | YES | YES | YES | | YES |
| Observations | 52,821 | 52,821 | 52,821 | | 52,821 |
| Number of IDs | 26,174 | 26,174 | 26,174 | | 26,174 |
| R-squared | 0.161 | 0.162 | 0.164 | | 0.164 |

*Note.* Same as Table 4.

**Supplementary Table 7. Education Level of the Insured and Their Family Caregivers, All Applicants in City X between Jul. 2017 and Sep. 2020**

| Education Level of the Insured | Education Level of Family Caregivers | | | Total |
| --- | --- | --- | --- | --- |
|  | Illiterate | Primary and Secondary School | High School and Above |  |
| Illiterate | 108(3.25%) | 2,509(75.62%) | 701(21.13%) | 3,318(100%) |
| Primary and Secondary School | 186(3.03%) | 4,356(70.96%) | 1,597(26.01%) | 6,139(100%) |
| High School and Above | 28(1.61%) | 901(51.72%) | 813(46.67%) | 1,742(100%) |
| Total | 322(2.88%) | 7,766(69.35%) | 3,111(27.78%) | 11,199(100%) |

*Source:* Authors’ analysis of data from a follow-up survey of all LTCI applicants conducted between July 2021 and January 2022. Of 52457 applicants by Aug. 2021, 43143 of them (or their family members) were followed. The survey inquired about the applicants’ health status, caregivers, etc.

**Supplementary Table 8. Places of Ageing before LTCI by Education and Grade of Disability, All Applicants in City X between Jul. 2017 and Sep. 2020**

|  | Total  (N=26,124) | Home  (N=18,741) | | Institution  (N=7,383) | |
| --- | --- | --- | --- | --- | --- |
|  | N | N | % | N | % |
| Education | | | | | |
| Illiterate | 6,208 | 4,879 | 78.59 | 1,329 | 21.41 |
| Primary & Secondary School | 14,035 | 10,261 | 73.11 | 3,774 | 26.89 |
| High School and Above | 5,881 | 3,601 | 61.23 | 2,280 | 38.77 |
| Disability Grade | | | | | |
| Grade-One Heavy | 16,515 | 12,803 | 77.52 | 3,712 | 22.48 |
| Grade-Two Heavy | 9,384 | 5,816 | 61.98 | 3,568 | 38.02 |
| Grade-Three Heavy | 225 | 122 | 54.22 | 103 | 45.78 |

**Supplementary Note 1. Assessment Procedure and Criteria of Grade of Disability**

In the first step of the assessment, three first-level evaluation indicators of activity of daily living, mental status, and sensory, communication, and social involvement determined each applicant’s classification of grade of disability.

First, the first-level indicator of activity of daily living was further determined by ten second-level evaluation indicators, with each measuring the applicant’s ability in a particular daily activity, such as eating, bathing, toileting, etc. For a particular daily activity, if the applicant could not perform it (nearly) completely, the corresponding second-level indicator would be assigned a value of zero. If she needed some but not a lot of help, the second-level indicator would be assigned a value of five. If she could do it (nearly) independently, the second-level indicator would be assigned a value of ten. The values of these ten second-level indicators added up to the value of the first-level indicator of activity of daily living (thus with a value between 0 and 100). According to this value of the first-level indicator, the applicant was classified as without impairment, with mild impairment, with moderate impairment, and with heavy impairment in the activity of daily living if the value was 100, between 65 and 95, between 45 and 60, and less than or equal to 40 respectively.

Second, the first-level indicator of mental status was further determined by three second-level indicators, with the first one measuring one’s basic cognitive ability by two simple questions, the second one measuring one’s severity of dementia, and the third one measuring one’s frequency of abnormal behaviors. The first second-level indicator would be assigned a value of two if the applicant answered both questions correctly, a value of one if only one answer was right, and a value of zero if both answers were wrong. The value of the second second-level indicator measuring one’s severity of dementia was determined by the total scores of twelve questions regarding one’s ability in memory, judgement, execution, communication, understanding, etc. For each one of these twelve questions, the score would be zero if the answer was totally negative, one if only partially negative, and two if totally positive. The scores of these twelve questions added up to the value of the second-level indicator measuring one’s severity of dementia (thus with a value between zero and twenty-four). The value of the third second-level indicator measuring one’s frequency of abnormal behaviors was determined by the total score of five questions regarding the frequency of five abnormal behaviors, including wandering around, talking to oneself, feeling paranoid, etc. For each question regarding a particular abnormal behavior, the score would be zero if the abnormal behavior (nearly) always happened, one if sometimes, and two if never. The scores of these five questions added up to the value of the second-level indicator measuring one’s frequency of abnormal behavior (thus with a value between zero and ten). Note that only when one’s value of the first second-level indicator measuring basic cognitive ability was zero or she could provide direct proofs on dementia from some A-grade tertiary hospital would the second and third second-level indicators begin to be assessed. For an applicant, she would be classified as without impairment and with mild impairment in mental status if her value of the second-level indicator measuring basic cognitive ability was two and one respectively, and would be classified as with moderate impairment in mental status if her value of the second-level indicator measuring basic cognitive ability was zero but the sum of the two remaining second-level indicators measuring severity of dementia and frequency of abnormal behavior was larger than ten. Otherwise, she would be classified as with heavy impairment in mental status.

Third, the first-level indicator of sensory, communication, and social involvement was determined by three second-level indicators measuring one’s vision, hearing, and social communication respectively. The values of these three second-level indicators would be three, two, one or zero depending on how well the applicant’s vision, hearing, and social communication was, and they added up to the value of the first-level indicator of sensory, communication, and social involvement (thus with a value between zero and nine). According to this value of the first-level indicator, the applicant was classified as without impairment, with mild impairment, with moderate impairment, and with heavy impairment in sensory, communication, and social involvement if the value was nine, between five and eight, between one and four, and zero respectively.

If the applicant was classified as with heavy impairment in activity of daily living, she would be directly classified as with heavy disabilities. The further classification within those with heavy disabilities was determined by one’s mental status and sensory, communication, and social involvement. If the applicant was further with heavy impairment both in mental status and sensory, communication, and social involvement, she would be classified as with grade-three heavy disabilities; if the applicant was further with moderate or heavy impairment in mental status *and* with moderate or heavy impairment in sensory, communication, and social involvement (but not both with heavy impairment), she would be classified as with grade-two heavy disabilities; the remaining with heavy disabilities would be classified as with grade-one heavy disabilities.

**Supplementary Note 2. Comparison between DID and Event Study**

Considering the data structure in Supplementary Table 3, we adopted a multi-period DID approach. Denote the average outcome as $\bar{Y}$, the treatment group as $T$, the control group as $C$, the time period before a policy shock as period 0 and the time period after that as period 1. Regression analysis with the standard DID method yields the following estimate of the average policy impact,

$${(\bar{Y}}_{1}^{T}-\bar{Y}_{0}^{T})-{(\bar{Y}}_{1}^{C}-\bar{Y}_{0}^{C}).$$

The second term in the bracket ${(\bar{Y}}_{1}^{C}-\bar{Y}_{0}^{C})$ represents the time trend between period 1 and period 0. On the other hand, standard event study yields the following estimate of the average policy impact

$${(\bar{Y}}_{1}^{T}-\bar{Y}_{0}^{T}).$$

The DID and the event study approaches yield identical estimates when the time trend term ${(\bar{Y}}_{1}^{C}-\bar{Y}_{0}^{C})$ is zero. This often happens when the time interval between period 0 and period 1 is short and thus any time trend is minimal.
